# Cell therapies for joint repair

**DOI:** 10.1186/1476-9255-12-S1-O7

**Published:** 2015-04-16

**Authors:** Cosimo De Bari

**Affiliations:** 1 University of Aberdeen, Regenerative Medicine Group, Musculoskeletal Research Programme, Institute of Medical Sciences, Aberdeen, AB25 2ZD, UK

## 

Arthritis, with osteoarthritis (OA) accounting for most of this burden, is a leading cause of disability in the western world. Symptomatic full-thickness defects of the knee joint surface require surgical treatment for symptom relief and to prevent secondary OA. Current gold standard of cell therapy for joint surface repair consists of implantation of autologous, culture-expanded articular chondrocytes underneath a periosteal flap or a synthetic membrane. Twenty years’ follow-up has proved autologous chondrocyte implantation (ACI) to be an effective and durable solution for the treatment of large full-thickness cartilage lesions of the knee joint. Articular chondrocytes, however, are difficult to expand because of their limited proliferative capacity and rapid de-differentiation *in vitro*. The use of mesenchymal stem cells (MSCs) as chondrocyte substitutes in an ACI-equivalent procedure is intensely pursued because MSCs are easy to isolate from bone marrow or other connective tissues such as adipose tissue and to expand in culture, and they have ability to form cartilage and bone. Clinical studies are ongoing to demonstrate non-inferiority of MSCs in structural and clinical outcomes when compared with chondrocytes. In addition to the use of *ex vivo* manipulated MSCs, an attractive approach to the repair of the joint surface is the activation of intrinsic repair mechanisms by targeting the stem cells naturally present in the joint. In this respect, several joint-associated tissues such as synovium, fat pad, periosteum, bone marrow, and even the articular cartilage itself, are known to contain cells that, after isolation and culture expansion, display properties of MSCs. Recently, by combining a double nucleoside labelling scheme with a clinically relevant mouse model of joint surface injury, we identified endogenous resident MSCs in the knee joint synovium *in vivo*. We are currently studying the functional roles of endogenous MSCs in joint health and diseases. Understanding the way stem cells function at their niche sites will help develop novel treatment approaches aiming at activating repair mechanisms to achieve tissue healing and restore joint homeostasis.

